# Graphene–PbS Quantum Dot Heterostructure for Broadband Photodetector with Enhanced Sensitivity

**DOI:** 10.3390/s24175508

**Published:** 2024-08-26

**Authors:** Jincheng Qing, Shicai Wang, Shuyi Gu, Lin Lin, Qinpei Xie, Daming Li, Wen Huang, Junxiong Guo

**Affiliations:** 1 School of Electronic Information and Electrical Engineering, Institute of Advanced Study, Chengdu University, Chengdu 610106, China; qingjincheng@stu.cdu.edu.cn (J.Q.); gushuyi@stu.cdu.edu.cn (S.G.); cdu1467337756@163.com (Q.X.); cdu085406ldm@163.com (D.L.); 2 School of Integrated Circuit Science and Engineering (Exemplary School of Microelectronics), University of Electronic Science and Technology of China, Chengdu 611731, China; wangshicai123@outlook.com (S.W.); linlin@std.uestc.edu.cn (L.L.); uestchw@uestc.edu.cn (W.H.); 3 School of Materials and Energy, University of Electronic Science and Technology of China, Chengdu 610054, China; 4 Chengdu Research Institute of UESTC, Chengdu 610207, China

**Keywords:** graphene, PbS quantum dots, broadband photodetector, high sensitivity, heterostructure

## Abstract

Photodetectors converting light into electrical signals are crucial in various applications. The pursuit of high-performance photodetectors with high sensitivity and broad spectral range simultaneously has always been challenging in conventional semiconductor materials. Graphene, with its zero bandgap and high electron mobility, is an attractive candidate, but its low light absorption coefficient restricts its practical application in light detection. Integrating graphene with light-absorbing materials like PbS quantum dots (QDs) can potentially enhance its photodetection capabilities. Here, this work presents a broadband photodetector with enhanced sensitivity based on a graphene–PbS QD heterostructure. The device leverages the high carrier mobility of graphene and the strong light absorption of PbS QDs, achieving a wide detection range from ultraviolet to near-infrared. Employing a simple spinning method, the heterostructure demonstrates ultrahigh responsivity up to the order of 10^7^ A/W and a specific detectivity on the order of 10^13^ Jones, showcasing significant potential for photoelectric applications.

## 1. Introduction

Photodetectors, which convert light into electrical signals, are integral components in various applications such as optical communication, imaging systems, environmental monitoring, and biological sensing [1,2,3]. The quest for high-performance photodetectors has been a driving force in the advancement of optoelectronic technology [4]. The ideal photodetector should exhibit a combination of high sensitivity and broad spectral range, attributes that are not easily achieved simultaneously with conventional materials and designs.

Recent emerging two-dimensional materials, such as graphene and transition-metal dichalcogenides, have demonstrated their great potentials for photodetection due to their unique optical and electronic properties [5,6,7]. Graphene, especially, possesses a zero bandgap nature that endows it with a theoretically unlimited absorption spectrum, covering from ultraviolet to infrared wavelengths [8]. This broad absorption profile makes graphene an attractive material for photodetectors that require operation across multiple spectral bands. Moreover, it also possesses an exceptionally high electron mobility, which is crucial for the rapid transport of photogenerated carriers [9]. However, the low light absorption coefficient in pristine graphene, stemming from its non-polar nature and the absence of a bandgap, poses a significant challenge for its use in efficient photodetection. The absorption of light in graphene is relatively weak, with a reported value of only about 2.3% for visible light [10]. This limitation restricts the generation of photocurrent and thus the sensitivity of graphene-based photodetectors.

To address this issue, researchers have explored various strategies to enhance the light–matter interaction in graphene [1]. One promising approach involves the integration of graphene with other low-dimensional materials that have strong light-absorbing properties [11,12]. Among diverse candidates, quantum dots (QDs), semiconductor nanocrystals with dimensions measuring a few nanometers, have been identified as an ideal material to complement graphene [11,13,14]. QDs exhibit size-dependent bandgaps, allowing for tunable absorption spectra, unique electrical and mechanical properties that can be tailored to specific applications, such as quantum edge sensors and processors [15]. Moreover, their large surface-to-volume ratio and strong confinement effects result in high extinction coefficients, which significantly enhance light absorption [16]. PbS QDs, in particular, have garnered significant interest due to their strong absorption coefficient and the possibility of bandgap engineering to cover a wide range of the solar spectrum [13]. To date, various works have investigated the integration of graphene with PbS QDs for optoelectronic applications, particularly in photodetectors and phototransistors [11,17,18,19,20,21,22]. These studies demonstrate the synergetic effect of combining the high mobility of graphene with the strong light absorption capabilities of PbS QDs, promisingly overcoming the limitations of both individual components. Several studies highlight the fabrication of hybrid graphene–PbS QD devices, reporting high responsivity up to 10^8^ A/W and detectivity reaching 10^12^ Jones, surpassing pristine graphene-based devices due to enhanced charge transfer and reduced recombination rates [18,22]. The performance of these devices is tuned by varying parameters such as QD size, film thickness, and gate voltage, revealing their impact on spectral response and time response. Additionally, lithography-based patterning techniques are employed to achieve high-resolution PbS QD/graphene photodetector arrays, facilitating their integration into semiconductor technology [18]. A memory function is introduced in some phototransistors by incorporating graphene oxide storage layers, expanding their applicability [21]. Vertical architecture phototransistors are also explored [11], showing potential for large-area, flexible, and low-cost infrared photodetectors with improved photoconductive gain mechanisms attributed to trap states. Although these important progresses have been achieved, the goal of high sensitivity, including responsivity and specific detectivity, has remained the pursuit of researchers.

In this paper, we demonstrate that the decoration of graphene with PbS QDs can be achieved through a simple spinning method for broadband photodetection. The heterostructure is designed to leverage the complementary properties of graphene and PbS QDs to achieve high sensitivity and a wide detection range from ultraviolet to near-infrared wavelengths. The fabrication process involves the decoration of graphene with PbS QDs achieved through a layer-by-layer deposition process, ensuring a uniform and stable integration. The resulting hybrid structure is then characterized, including absorption spectroscopy, atomic force microscopy (AFM), and Raman spectroscopy, to evaluate its optical and structural properties. The photodetection performance of the PbS QD-decorated graphene devices is also assessed through electrical and optical measurements. The current–voltage (*I*–*V*) characteristics under dark and illuminated conditions provide insights into the device’s operating mechanism and the influence of QDs on the electrical properties of graphene. We show that the fabricated device possesses an ultrahigh responsivity up to an order of 10^7^ A/W and a highest specific detectivity on the order of 10^13^ Jones. The findings of this study contribute to the understanding of the interaction between the interfaces of graphene and PbS QDs. The results highlight the potential of the integrated PbS QDs/graphene structure to achieve high sensitivity and a broad spectral response, making it a strong contender for photoelectric applications.

## 2. Materials and Methods

### 2.1. Materials

The single-layer graphene grown on copper substrate using chemical vapor deposition (CVD) method was purchased from the Shenzhen Six Carbon Technology Co., Ltd., Shenzhen, China. The PbS QDs (with diameter of ~4.5 nm) covered with a ligand of oleate were provided by the Xingzi SciTech Co., Ltd., Shanghai, China. The reagents of polymethyl methacrylate (PMMA), iron chloride (FeCl_3_), toluene, actone, isopropyl alcohol (IPA), 1,2-ethanedithiol, acetonitrile, and absolute alcohol were purchased from the Chengdu Keweizhuo Technology Co., Ltd., Chengdu, China, without further purification. The deionized water was prepared with a KZ-ZDX-20L ultra-pure water machine (Shanghai Keye Co., Ltd., Shanghai, China).

### 2.2. Device Fabrication

The CVD-grown graphene was transferred onto the surface-cleaned SiO_2_/Si substrate following our previous wet transfer method [2]. Briefly, the wet transfer process of graphene involves several meticulous steps to ensure a clean and efficient transfer onto a target substrate, and the details are listed as follows. (1) Preparation of substrate: The first step involves preparing the recipient substrate, cleaned thoroughly using solvents with IPA and acetone to remove any contaminants or residues. (2) Support layer: A support layer, made of PMMA, was spin-coated onto the graphene layer. (3) Etching: The copper substrate was etched away using 1 mol/L FeCl_3_ solution, leaving behind the PMMA-supported graphene floating on the solution. (4) Floating transfer: The PMMA-supported graphene was then carefully lifted off and floated onto a water bath or directly onto the surface of the pre-prepared SiO_2_/Si substrate. Great care is taken to avoid wrinkles or tears. (5) Drying: The assembly was allowed to dry naturally or under controlled conditions to evaporate the water without inducing stress that could damage the graphene layer. (6) Removal of PMMA: Once dry, the PMMA layer was removed using acetone, which does not affect the graphene or the underlying substrate.

The transferred graphene was then patterned using a MicroWriter ML3 direct-write photolithography machine (Durham Magneto Optics, Cambridge, UK) combined with the Phantom Reactive Ion Etcher with Inductively Coupled Plasma Source (Trion Technology, Clearwater, FL, USA). The patterned graphene was then deposited with Au/Ni contacts. The fabricated devices were subjected to an annealing process under a controlled atmosphere in a 100 standard-cubic-centimeter-per-minute (sccm) argon (Ar)/hydrogen (H_2_) gas flow (volume ratio of 9:1) at 200 °C for 2 h to improve adhesion, remove residual impurities, and enhance electrical properties. Finally, the PbS QDs were dissolved in toluene at a concentration of 25 mg/mL and deposited on patterned graphene to complete the device fabrication.

### 2.3. Characterization and Measurements

Optical images were captured employing a BX51M microscope (OLYMPUS, Tokyo, Japan). High-resolution examination of device components was conducted via a JSM 7500F scanning electron microscope (JEOL, Tokyo, Japan), operated at an acceleration voltage of 15 kV. Raman spectroscopy was facilitated by employing a tip-enhanced Raman scattering (TERS) technique. Our experimental setup was comprehensively equipped with an integrated scanning probe microscope coupled with a Raman micro-spectrometer (HORIBA, Yokohama, Japan). The excitation laser for Raman characterizations was set at a wavelength of 532 nm. The photoelectric performances were recorded using a CS series semiconductor parameter (Precise, Wuhan, China) combined with diode lasers (Changchun New Industries Optoelectronics Technology Co., Ltd., Changchun, China). The incident laser sources, including Raman characterization and photodetection measurements, were controlled with power below 1 mW to avoid the heating effect. All characterization and measurement processes were conducted at ambient temperature.

## 3. Results and Discussion

### 3.1. Fabrication and Architecture of PbS QD/Graphene Heterostructure-Based Photodetector

**Figure 1:**
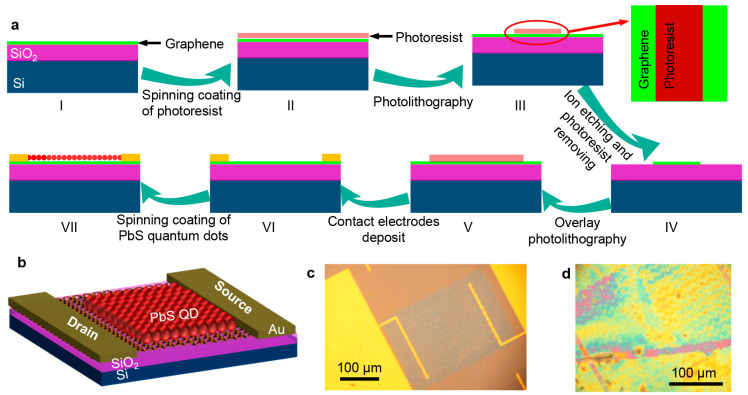
Conceptual design of PbS QDs/graphene heterostructure-based photodetector. (**a**) Schematic of device fabrication process. The right panel of step III is the top view of patterned photoresist. Steps from I to IV are the cross-view of device and the steps from V to VII are the side-view of device. (**b**) Three-dimensional model of the designed photodetector. Optical images of fabricated devices based on bare graphene (**c**) and after decorating PbS QDs (**d**).

The fabrication process, as shown in Figure 1a, starts with transferring graphene onto a SiO_2_/Si substrate using a wet transfer method (step I), followed by etching into a 50 × 120 μm^2^ rectangular shape (Steps II to IV), and the details can be found in the Materials and Methods section. Nickel/gold electrodes are deposited using magnetron sputtering (Steps V and VI), and finally, PbS QDs are spin-coated onto graphene (Step VII). The PbS QDs are dissolved in toluene at a concentration of 25 mg/mL. Layer-by-layer deposition and ligand exchange techniques are employed during the spin-coating process to improve detector stability and carrier transport. The spin-coating procedure includes depositing the oil-soluble PbS QDs on graphene, followed by a layer of the diluted 1,2-ethanedithiol solution, repeating these steps three times. The ligand exchange replaces long chains with short ones, using 1,2-ethanedithiol diluted to a 2% solution in acetonitrile.

The proposed device structure is depicted in Figure 1b, where SiO_2_ acts as the gate oxide layer, Si as the gate electrode, graphene as the conductive channel, and PbS QDs as the absorption layer. The structure of the fabricated photodetectors based on bare graphene and graphene–PbS QDs is presented in Figure 1c and Figure 1d, respectively.

**Figure 2:**
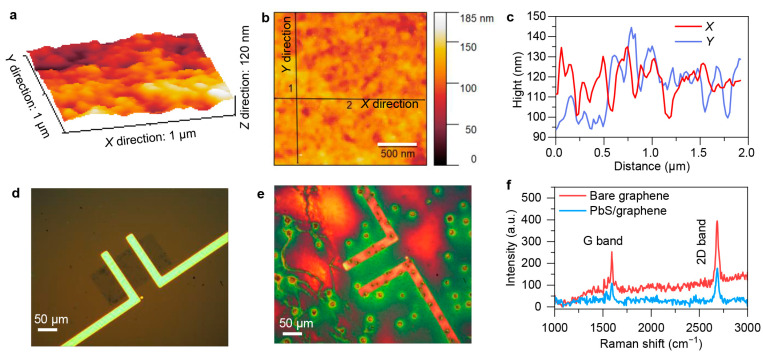
Structure characterization of PbS QDs/graphene heterostructure-based photodetector. Three-dimensional (**a**) and two-dimensional (**b**) views of AFM height characterizations for PbS QDs/graphene heterostructure active layer. (**c**) Height changing with X- and Y-direction distances, which is extracted from panel (**b**). Overall view of optical image (**d**) and corresponding Raman mapping image (**e**) of fabricated device under Raman characterization. (**f**) Raman shift curves of device based on bare graphene and after decorating PbS QDs.

We now proceed to analyze the morphology of the device after the deposition of QDs. Figure 2a–c display the atomic force microscopy (AFM) images and corresponding height distribution of the fabricated device. A region of 1 μm × 1 μm was selected for measurement and then scanned to obtain 256 × 256 pixel points with a resolution of approximately 4 nm. From the morphology diagram, we observe that the root mean square of the height in this area is 11.17 nm (approximately two to three layers of PbS QDs), indicating that the device has relatively minor undulations in height, and hence the QDs spread by spin-coating are relatively uniform.

To confirm the deposit of the PbS QDs onto the graphene, Raman characterizations were also performed. The Raman spectrum of graphene is primarily composed of three characteristic peaks: D band (~1350 cm^−1^), G band (~1580 cm^−1^), and 2D band (~2700 cm^−1^) [23,24,25]. The number of graphene layers can be estimated by using the intensity ratio of the G and 2D bands [26]. Here, the transferred graphene without further etching shows two distinct characteristic peaks of the G band at 1588.2 cm^−1^ and 2D band at 2696.4 cm^−1^, as shown in Figure S1 of the Supplementary Materials. The full width at half maximums (FWHMs) are 26.2 and 43.2 cm^−1^ in the G and 2D bands, respectively. We roughly calculated that the integrated intensity ratio of these two bands (*I*_G_/*I*_2D_) is approximately 0.31, suggesting that transferred graphene is monolayer [27,28,29]. The details of the graphene layers estimation are provided in Supplementary Note S1 and Figures S2–S4 of the Supplementary Materials. Meanwhile, the intensity ratio of the Raman G and 2D bands can also effectively reflect the doping situation. The larger the ratio, the lower the electron concentration at the Fermi level in graphene, and the closer it is to intrinsic graphene [2,23]. That is to say, the higher the ratio, the lower the carrier concentration in graphene. From Figure 2d–f, after spin-coating with QDs, we can partly conclude that the electron concentration in graphene decreases, the resistance of graphene increases, and the Fermi level will be lowered, leading to a leftward shift in the graphene Dirac point voltage.

### 3.2. Optical and Electrical Performance of Fabricated Photodetector

**Figure 3:**
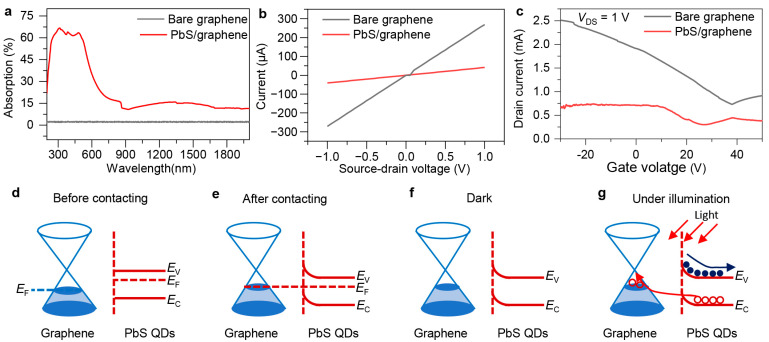
Optical and electrical characterization of the fabricated photodetector. Absorption (**a**), source–drain current as a function of bias voltage (**b**), and transfer characterization (**c**) of devices based on bare graphene and after decorating with PbS QDs. Schematic of band alignment in device based on graphene before (**d**) and after (**e**) contacting with PbS QDs. Schematic of band alignment in device based on PbS QD/graphene heterostructure under dark (**f**) and illumination (**g**) conditions.

Before conducting photoelectric measurements of the fabricated device, PbS QDs before and after deposition were first characterized by using a UV-vis-NIR spectrophotometer (Agilent Technologies, Santa Clara, CA, USA). The absorption spectrum of bare PbS QDs (Figure S5) shows its optical response can be extended to near infrared exceeding 1350 nm. As shown in Figure 3a, the absorption of pure graphene on the SiO_2_/Si substrate is only approximately 2%, while the PbS QD/graphene on SiO_2_/Si structure exhibits high light absorption, showing broad-spectrum absorption across ultraviolet to near-infrared regions. The absorption rate generally shows a trend of gradually decreasing and then stabilizing as the wavelength increases.

Figure 3b shows the *I*-*V* curve obtained from the fabricated device operating at zero gate voltage. The current exhibits a good linear relationship with voltage, indicating that the prepared source and drain electrodes form a good ohmic contact with the graphene. Meanwhile, according to the curve, the dark current of the single graphene is very high, with a channel resistance of about 1000 Ω. The issue of high dark current also severely affects the performance of the graphene photodetector. In comparison, the output curve after spin-coating the PbS QDs is represented by the red curve, where the current still shows a linear change with voltage, indicating that the spin-coating of QDs has not affected the contact electrodes of the device, and it still exhibits good ohmic contact. Moreover, after spin-coating the PbS QDs, the dark current of the device is significantly reduced. The analysis suggests that the reason for the decrease in dark current is the electron–hole recombination between graphene and PbS QDs [30], which leads to a reduction in the carrier concentration in the graphene channel.

We know that graphene is a zero bandgap material, which means it has relatively special transfer characteristics. Figure 3c shows a comparison of the transfer curves of graphene before and after spin-coating with PbS QDs. The device has a channel width of 50 μm, a length of 80 μm, and the source–drain voltage tested is 1 V. The gate voltage (*V*_G_) is scanned from −30 V to +50 V. Before spin-coating the QDs, as *V*_G_ increases, the source-drain current (*I*_DS_) first decreases and near the end increases. The gate voltage corresponding to the minimum drain current is called the Dirac point voltage (*V*_D_), i.e., the electrical neutrality point. For intrinsic graphene, theoretically *V*_D_ = 0 V, but the graphene prepared in this paper has a *V*_D_ that is not zero, indicating that the graphene is doped. Before spin-coating the QDs, the *V*_D_ corresponding to the Dirac point is about 36 V, and after spin-coating the QDs, the *V*_D_ corresponding to the Dirac point is about 25 V. Therefore, after spin-coating, the Dirac point of graphene moves to the left, corresponding to a decrease in the Fermi level. When *V*_G_ < *V*_D_, graphene is hole transport, and when *V*_G_ > *V*_D_, graphene is electron transport. Theoretically, the transfer curve of graphene is symmetrical, but the transfer curve prepared in this paper is not symmetrical. The reason may be due to the imbalanced carrier injection between the source–drain electrodes [31]. Also, it can recognize that this reduction in *V*_D_ corresponding to Dirac point is related to the dopants or impurities of graphene (Figure 2) [9]. The impurity-induced graphene electronic damage could be reduced by some auxiliary methods such as natural drying and etching to remove the residual contaminants [32,33].

According to the transfer characteristic curve, we can calculate the mobility of graphene, thereby determining the impact of the PbS QDs on the electrical conductivity of graphene. The mobility of graphene is given by Equation (1), where *L* is the length of the graphene channel, *W* is the channel width, and *C* is the capacitance of the SiO_2_ layer [9]. Here, the thickness of SiO_2_ is 300 nm, corresponding to a capacitance of 1.2 × 10^−8^ F/cm^2^, and *V*_*DS*_ is the source–drain voltage. According to the transfer curve, the carrier mobility of graphene decreases after spin-coating with QDs, possibly because the deposition process of the QDs caused damage to the graphene. After calculation, the carrier mobility of graphene before and after spin-coating is 2133.3 cm^2^ V−^1^s−^1^ and 1600 cm^2^ V−^1^s−^1^, respectively. The reason of the Dirac point shift is closely related to the graphene behaviors, as described in the Raman characteristics mentioned above. The reduced carrier mobility of graphene after decorating the PbS QDs is also related to the additional impurity during the production of the photodetectors [32,33].

(1) $$\mu =\frac{\partial I_{DS}}{\partial V_{G}}\frac{L}{WCV_{DS}}$$

To better understand the impact of PbS QDs on graphene, the energy band structures of both PbS QDs and graphene were analyzed. Figure 3d shows the energy band diagram of PbS QDs and graphene before contact. The Fermi levels of the two materials are not the same; the conductive carriers in graphene are holes, while the conductive carriers in PbS QDs are electrons. After contacting the PbS QDs with graphene, the energy band structure changes, as shown in Figure 3e. Upon contact, the holes in graphene recombine with the electrons in the PbS QDs, causing them to reach the same Fermi level, ultimately forming an internal electric field on the surface.

The working principle of the device is mainly as follows. When there is no light illumination, the graphene and PbS QDs are in thermal equilibrium. At this time, electrons on the PbS QD side cannot move to the graphene side, and the holes in the QDs cannot transport to the PbS QD side, as shown in Figure 3f. When light is incident on the PbS QDs, the QDs absorb photons to generate electron–hole pairs. Under the action of the built-in electric field formed between the QDs and graphene, the holes are transported to the graphene layer. Under the influence of the external electric field, they move towards the electrodes, eventually forming an electric current. The energy band diagram is shown in Figure 3g. The holes move to the graphene layer, forming p-type doping, while the electrons are captured in the QDs layer.

### 3.3. Photodetectrion Performance of Fabricated Device

We turn to explore the output characteristics of the fabricated photodetectors, and all measurements were conducted with zero-gate voltage. The data were collected by measuring five devices that are fabricated with a same method. As above mentioned, we have used 1,2-ethanedithiol diluted to a 2% solution in acetonitrile to exchange the ligand long chains with short ones. As shown in Figure S6a, it demonstrates the photoresponse of the device without ligand exchange, where a weak on–off photoresponse is observed because of its hindering carrier transport in PbS capped with long-chain oleate. After ligand exchange, photogenerated carriers can be transported to the graphene layer and subsequently collected by the electrode. In comparison, it presents a device prepared using an additional ligand exchange step, showing a significant on–off photoresponse (Figure S6b).

**Figure 4:**
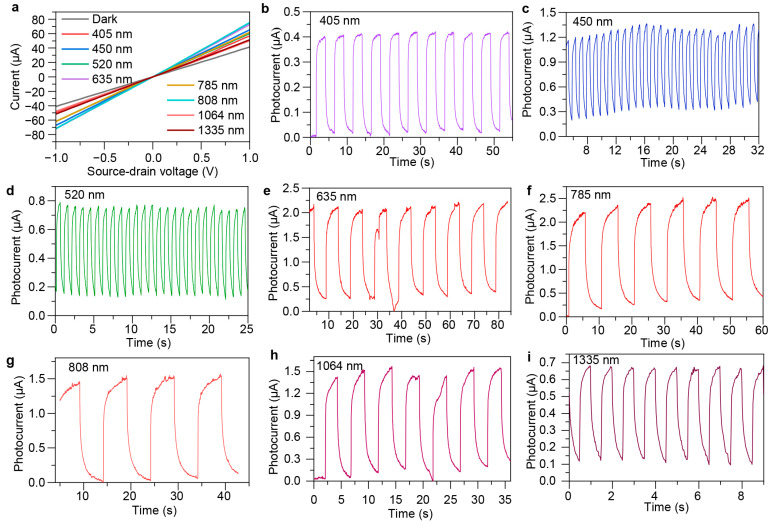
Photoelectric characterization of the fabricated device. (**a**) Source–drain current as a function of bias voltage under dark condition and different incident wavelengths. (**b**–**i**) Photocurrent as a function of measurement time under different wavelengths from 405 to 1355 nm, demonstrating its broadband response in the fabricated device.

Figure 4a shows the current comparison curve of Device 4 under dark and light illumination with different wavelengths. From the *I*–*V* curves, it can be seen that the photodetector belongs to the photoconductive type, and there is a good ohmic contact between graphene and the source–drain electrodes, with the light and dark current levels in the microampere range. Under illumination of different laser incident wavelengths, due to different incident light power and different light absorption rates of PbS QDs at different wavelengths, the current under light illumination is different. The device exhibits a distinct photocurrent at 405, 450, 520, 635, 785, 808, 1084, and 1335 nm wavelengths.

We further measured the transient photoresponse of the device with *V*_D S_ = 0.1 V. More quantitative information about the device response under pulsed laser illumination at the incident wavelengths from 405 to 1335 nm is shown in Figure 4b–i. These records were obtained with a light duty cycle of 50%, indicating that the device has a broadband spectral response from the visible light to the near-infrared regions. We observed that the device response time (period of photocurrent varying from 10% to 90%) varies at different wavelengths (Table 1). This is because the quantum dots have different light absorption at different wavelengths, and the incident light power is not the same, resulting in different amounts of photogenerated carriers [34].

**Figure 5:**
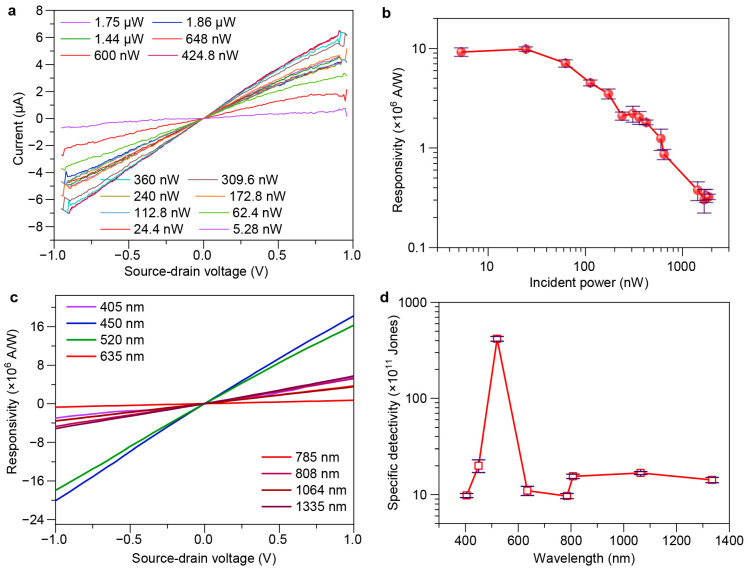
Detection performance of the fabricated device. (**a**) Source–drain current as a function of bias voltage under different incident power. (**b**) Responsivity as a function of incident laser power. (**c**) Responsivity as a function of bias voltage under different incident wavelengths. (**d**) Specific detectivity as a function of incident wavelengths. The error bars in (**b**,**d**) mean the standard deviation of responsivity and specific detectivity, respectively, obtained from 5 devices.

To explore the impact of light power on device performance, we also measured the output characteristics under different light powers, with the light source wavelength being 520 nm. Figure 5a shows the variation of photogenerated current with source–drain voltage under different light powers for the device. From the *I*–*V* curves, it can be concluded that at the same voltage, the photocurrent measured by the device varies with different incident light powers.

Responsivity (*R*_ph_) reflects the ability of a device to generate photocurrent under unit light power illumination. Under the conditions of an operating gate voltage of 0 V and a source–drain voltage of 0.1 V, the responsivity of the device under different light powers was calculated by *R*_ph_ = *I*_ph_/*P*_in_, where *P*_in_ is the incident power [2]. The calculation results under an incident wavelength of 520 nm are shown in Figure 5b,c. As the light power increases, the overall trend is to first increase and then decrease. The reason for the initial increase followed by a decrease is as follows: When the incident light power is low, the light absorption of the QDs has not reached saturation. As the incident light power increases, the more photons absorbed by the QDs, the more photogenerated carriers are produced, the larger the photocurrent, and the higher the response. When the incident light power reaches a certain value, the light absorption of the QDs becomes saturated and no longer increases with the increase in incident light power. Therefore, the photocurrent produced is constant, and the responsivity gradually decreases. Among them, the maximum responsivity is achieved at an incident light power of about 100 nW, with a maximum value that can reach up to order of 10^7^ A/W.

The specific detectivity (also known as the detectivity, D*) reflects a detector’s ability to detect weak signals. The specific detectivity of the devices in this paper was calculated using Equation (2), and the results are shown in Figure 5d, where *e*, *A*, and *I*_d_ represent the elementary charge, active area, and dark current, respectively [4]. The noises of fabricated devices were measured at room temperature in a dark environment with a semiconductor parameter (Precise CS series). It can be concluded that the detectors fabricated in this paper have a high specific detectivity, indicating a strong capability for detecting weak signals. Specifically, as the incident wavelength is 520 nm, the specific detectivity can reach approximately 4.18 × 10^13^ Jones (1 Jones = 1 cm⋅Hz^1/2^⋅W^−1^) with device operating at a gate voltage of 0 V and a source–drain voltage of 0.1 V.

(2) $$D^{*}=\frac{R_{ph}\sqrt{A}}{\sqrt{2eI_{d}}}$$

Finally, the device fabricated in this work was compared with those reported in the literature, and the results are shown in Table 2. Compared with the graphene–QD devices reported in the literature [11,18,19,21,22,35,36,37], although the experimental methods are similar and the photoelectric performances are not the best, the light absorption of the device in this work was greatly enhanced after spin-coating with PbS QDs. This leads to an increase in photogenerated carriers, resulting in a responsivity and specific detectivity that are comparable to, or even better than, the data reported in the literature.

## 4. Conclusions

In conclusion, we have presented a significant advancement in the development of high-performance photodetectors through the integration of PbS QDs with graphene. The resulting heterostructure has been demonstrated to offer superior sensitivity and a broad spectral response, overcoming the limitations of pristine graphene’s low light absorption. The strategic layer-by-layer deposition of PbS QDs onto graphene ensured a uniform and stable integration, which was crucial for enhancing the device’s photodetection capabilities. Characterization through absorption spectroscopy, AFM, and Raman spectroscopy confirmed the hybrid structure’s improved optical and structural properties. The photodetection performance, with an ultrahigh responsivity of up to 10^7^ A/W and a specific detectivity on the order of 10^13^ Jones underscores the device’s potential for high-speed and sensitive photodetection applications. The work also sheds light on the interface interactions between graphene and PbS QDs, revealing a leftward shift in the Dirac point voltage, indicative of reduced Fermi level and increased resistance in graphene. This shift, linked to the recombination of electrons and holes, is pivotal for the device’s enhanced photocurrent generation. Furthermore, the comparison with the existing literature shows that this work’s device outperforms or matches other graphene–QD-based photodetectors, validating the effectiveness of the integration approach. The findings not only contribute to the advancement of graphene-based photodetectors but also set a foundation for the future development of optoelectronic devices with improved performance for various applications.

## Figures and Tables

**Table 1:** Comparison of response time in device with different wavelengths.

Wavelength (nm)	Rise Time (s)	Fall Time (s)
405	0.21	0.91
450	0.5	0.5
520	0.1	1.2
635	1.3	1.6
785	1.3	1.8
808	1.9	1.8
1064	1.6	1.7
1335	1.5	1.3

**Table 2:** Comparison of photoelectric performance of current typical photodetectors using graphene combined with various quantum dots.

Device Architecture	Spectral Response	Highest Responsivity (A/W)	Highest Specific Detectivity (Jones)	Bias Voltage (V)	Ref.
SnO_2_ QDs/graphene	370 to 808 nm	9.6 × 10^2^	1.8 × 10^13^	−5	[35]
HgTe QDs/graphene/Si	405 to 880 nm 1500 to 2400 nm	0.9 0.3	1.5 × 10^10^ ND	1 0	[36]
Si QDs/graphene	300 to 1100 nm	0.495	7.4 × 10^9^	−1	[37]
PbS QDs/graphene	500 to 1400 nm	>10^8^	~10^12^	5	[18]
PbS QDs/graphene	400 to 1000 nm	(9.56 ± 0.20) × 10^3^	(8.8 ± 0.19) × 10^11^	1	[19]
PbS QDs/graphene	750 to 1350 nm	2 × 10^3^	7 × 10^12^	−3	[11]
PbS QDs/graphene	808 nm	33	2.2 × 10^8^	−2	[21]
PbS QDs/graphene	500 to 1350	>10^7^	ND	5	[22]
PbS QDs/graphene	405 to 1335 nm	(1.01 ± 0.05) × 10^7^	(4.18 ± 0.24) × 10^13^	0.1	This work

ND: No data.

## Data Availability

The original contributions presented in the study are included in the article/Supplementary Material; further inquiries can be directed to the corresponding author.

## Supplementary Materials

The following supporting information can be downloaded at: https://www.mdpi.com/article/10.3390/s24175508/s1, Supplementary Note S1: Estimation of graphene layer by using Raman spectrum; Figure S1: Raman characterization for graphene transferred onto SiO_2_:Si Substrate; Figure S2: Raman characterization for graphene and graphite; Figure S3: Characterization of graphene using high-frequency Raman spectra; Figure S4: Raman characterization for graphene with different layers; Figure S5: Absorption curve of bare PbS quantum dots; Figure S6: Transient photoresponse comparison of the device.
