# Supplementary material for: Graphene–PbS Quantum Dot Heterostructure for Broadband Photodetector with Enhanced Sensitivity
Source: Sensors (Basel). 2024 Aug 26;24(17):5508. doi: 10.3390/s24175508 (PMC11397984; doi:10.3390/s24175508)
Supplement: Supplementary file 1 [file sensors-24-05508-s001.zip › sensors-3138791-supplementary.pdf]

sensors

MDPI

Supporting Information for

Graphene–PbS Quantum Dot Heterostructure for Broadband Photodetector with Enhanced Sensitivity

Jincheng Qing [1,†], Shicai Wang [2,†], Shuyi Gu [1], Lin Lin [2], Qinpei Xie [1], Daming Li [1], Wen Huang [2] and Junxiong Guo [1,3,4,*]

[1] School of Electronic Information and Electrical Engineering, Institute of Advanced Study, Chengdu University, Chengdu 610106, China
[2] School of Integrated Circuit Science and Engineering (Exemplary School of Microelectronics), University of Electronic Science and Technology of China, Chengdu 611731, China
[3] School of Materials and Energy, University of Electronic Science and Technology of China, Chengdu 610054, China
[4] Chengdu Research Institute of UESTC, Chengdu 610207, China
† These authors contributed equally to this work.
* Correspondence: guojunxiong@cdu.edu.cn

Supplementary Note S1: Estimation of Graphene Layer by Using Raman Spectrum

Figure S1 shows the Raman spectrum of our transferred graphene without further etching. It exhibits two distinct characteristic peaks of G band at 1588.2 cm^{-1} and 2D band at 2696.4 cm^{-1}. The full width at half maximum (FWHM) are 26.2 and 43.2 cm^{-1} in G and 2D bands, respectively. The integrated intensities ratio of these two bands (I_G/I_{2D}) is approximately 0.31.

In the early stage of graphene investigation, Raman had been employed as a powerful tool to characterize the graphene layers. Figure S2 compares the Raman spectra of graphene and bulk graphite, using a laser of 514.5 nm as excitation source [1]. Two prominent features are observed: a peak at 1580 cm^{-1} (G band) and a band at 2700 cm^{-1} (2D band). Figure S2b exhibits significant differences in the shape and intensity of the peak between graphene and bulk graphite. The peak in bulk graphite comprises two components, 2D1 and 2D2, each approximately 1/4 and 1/2 the height of the peak, respectively. Graphene, on the other hand, features a single, sharp peak, roughly four times more intense than the peak.

More quantitative information about the intensity of graphene G and 2D band are shown in Figure S3. Three general observations about G band 2D bands can be made from Figure S3 [2]: (1) The G-band frequency decreases with increasing graphene layers n, particularly for small n values. (2) The second-order band at ~2700 cm^{-1} (2D band) displays an intriguing graphene layer-dependence in both shape and frequency. (2) When number of the graphene layers is less than 5, the 2D band is more sensitive than the G-band.

The next consideration is selecting the appropriate excitation laser. While any common Raman laser can successfully measure graphene, it's crucial to consider the substrate. Graphene is typically deposited on Si or SiO2, which may fluoresce with NIR lasers (780/785 nm). Thus, visible lasers, not only 514 or 633 nm, 532 nm is also usually recommended (such as RM5, Thermo Scientific DXR, HORIBA, etc).

Figure S4 shows the Raman spectra for graphene layers characterization with 532 nm excitation [3]. In Figure S4a, it compares the Raman spectra of single- and double-layer graphene labeled A and B. Notably, the Raman signal undergoes significant alterations upon peeling off the penultimate layer, with the G band experiencing a substantial decrease in intensity and shifting towards higher wave numbers. To facilitate comparisons between data from different flakes and measurement runs, it focuses on the ratio of integrated intensities of the G and D' (also labeled 2D) band, illustrated in Figure S4b. Most changes can be attributed to the decrease in the G band, as the spectral weight of the D' band remains relatively stable. The integrated intensity ratio exhibits an almost linear increase from one to four layers, suggesting that the number of graphene layers in our work is 1.

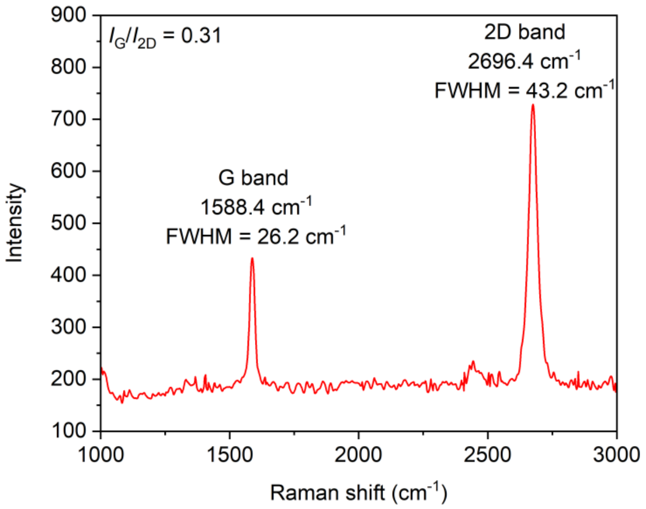

Figure S1. Raman characterization for graphene transferred onto SiO2:Si substrate.

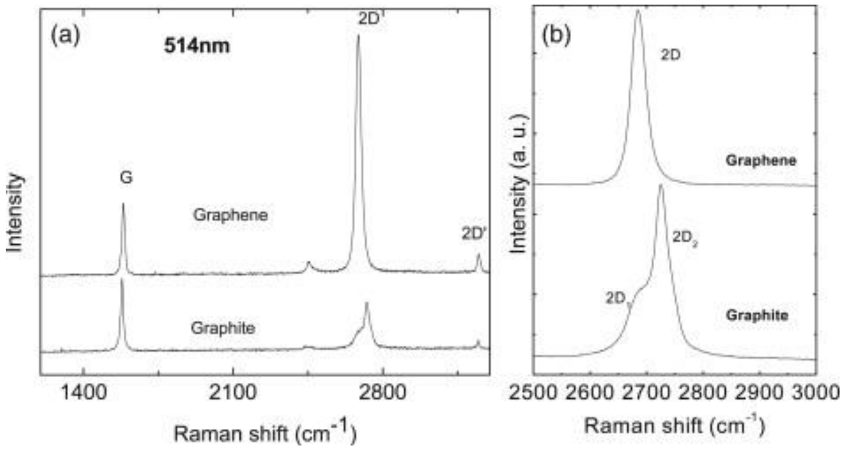

Figure S2. Raman characterization for graphene and graphite.
(**a**) Comparison of the Raman spectra of graphene and graphite. (**b**) Comparison of the 2D bands in graphene and graphite. The wavelength of the laser excitation was 532 nm. The figure is reprinted with permission from Ref. [1] (License Number 5844091500131). Copyright © 2007 Elsevier Ltd.

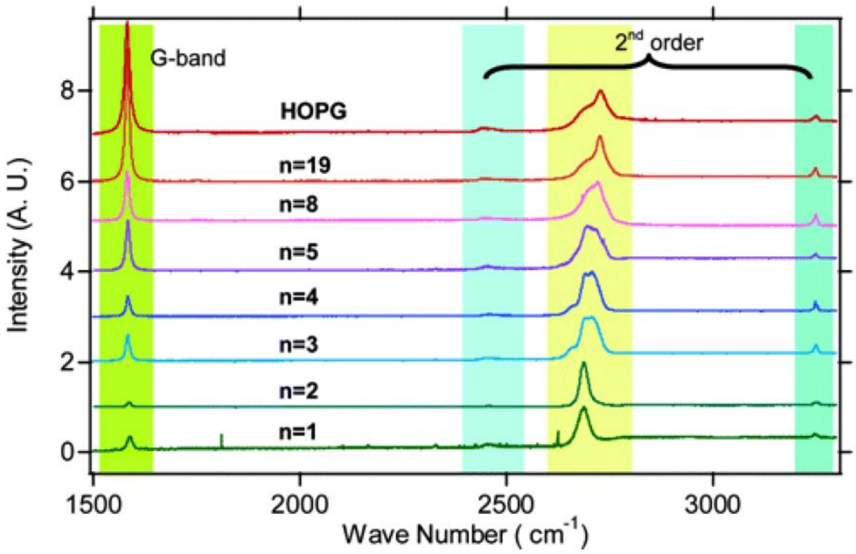

Figure S3. Characterization of graphene using high-frequency Raman spectra.
The spectra were collected using a SiO2/Si as graphene supporting layer. The wavelength of the laser excitation was 514 nm. The figure is reprinted with permission from Ref. [2]. Copyright 2006 American Chemical Society.

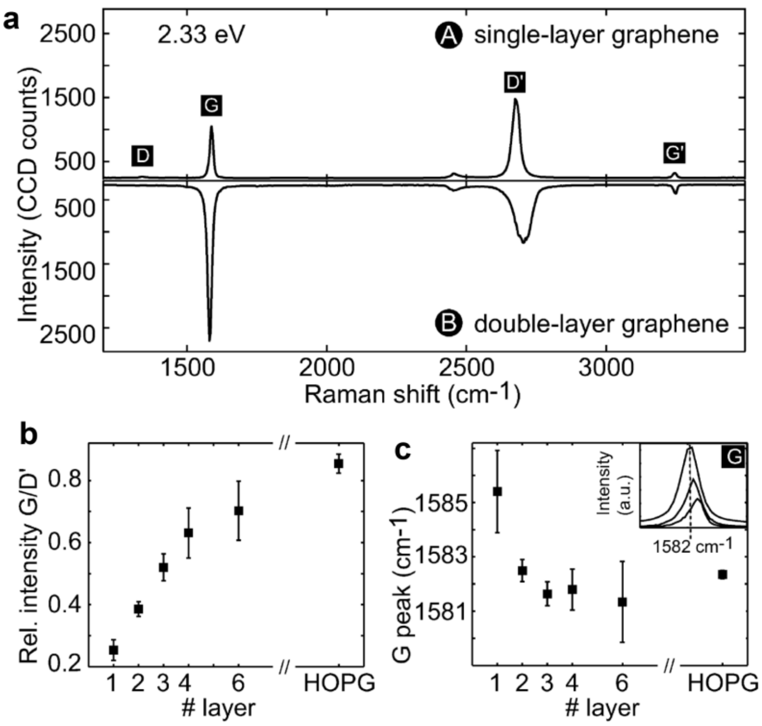

Figure S4. Raman characterization for graphene with different layers.
(**a**) Raman spectra of single- and double-layer graphene. (**b**) The integrated intensity ratio of G and D′ (also 2D) bands as a function of graphene layers. (**c**) The position of G band as a function of graphene layers. The inset of (**c**) represents the G band for graphite (upper), double- (middle) and single-layer (lower) graphene. The error bars in (**b**) and (**c**) are the standard deviations. The wavelength of the laser excitation was 532 nm. The figures (**a**) to (**c**) are reprinted with permission from Ref. [3]. Copyright 2007 American Chemical Society.

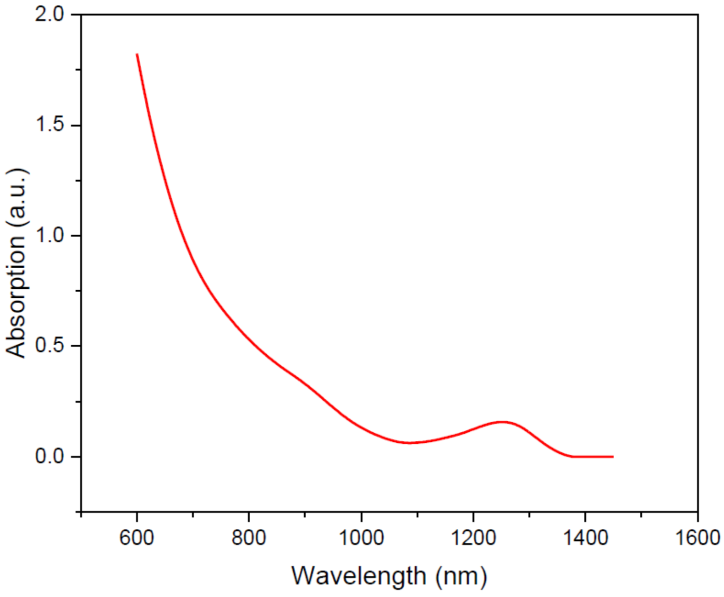

Figure S5. Absorption curve of bare PbS quantum dots.

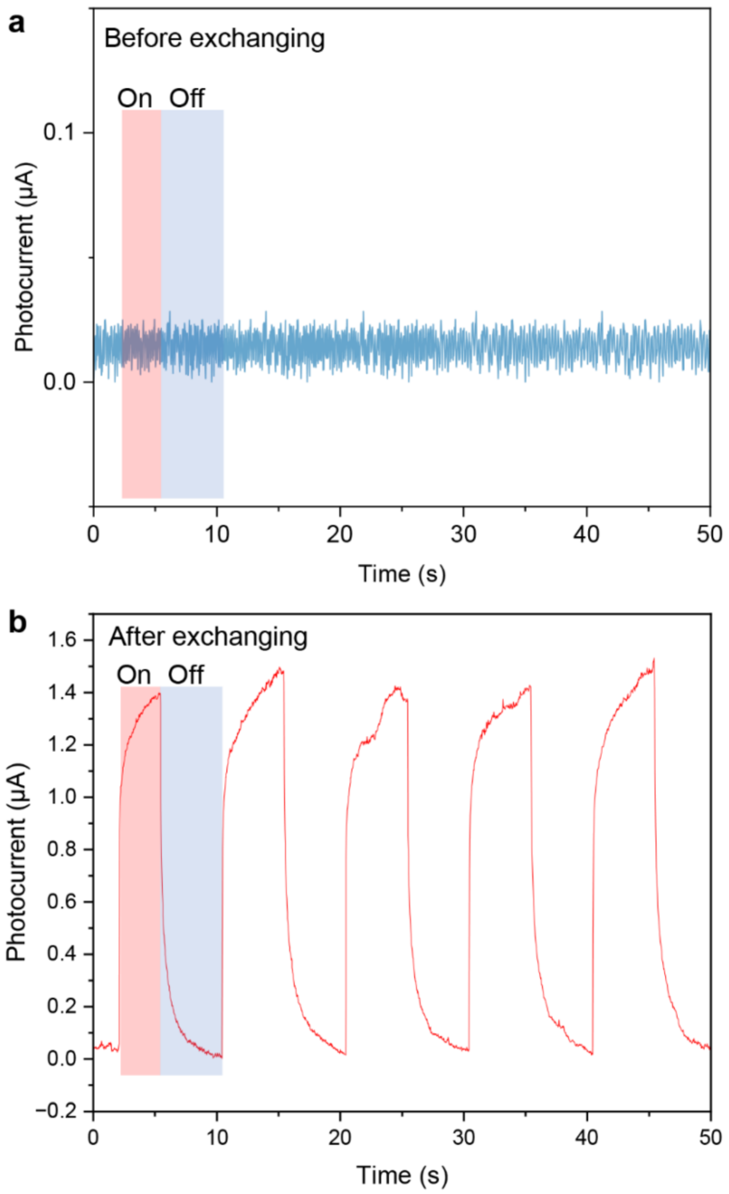

Figure S6. Transient photoresponse comparison of the device.
(**a**) Pristine PbS QDs capped with long-chain oleate. (**b**) Exchanging long-chain ligand using 1,2-ethanedithiol diluted to a 2% solution in acetonitrile.

References

1. Ferrari, A.C. Raman spectroscopy of graphene and graphite: Disorder, electron–phonon coupling, doping and nonadiabatic effects. *Solid State Commun.* **2007**, *143*, 47–57. https://doi.org/10.1016/j.ssc.2007.03.052.
2. Gupta, A.; Chen, G.; Joshi, P.; Tadigadapa, S.; Eklund. Raman scattering from high-frequency phonons in supported n-graphene layer films. *Nano Lett.* **2006**, *6*, 2667–2673. https://doi.org/10.1021/nl061420a.
3. Graf, D.; Molitor, F.; Ensslin, K.; Stampfer, C.; Jungen, A.; Hierold, C.; Wirtz, L. Spatially resolved Raman spectroscopy of single- and few-layer graphene. *Nano Lett.* **2007**, *7*, 238–242. https://doi.org/10.1021/nl061702a.

Disclaimer/Publisher's Note: The statements, opinions and data contained in all publications are solely those of the individual author(s) and contributor(s) and not of MDPI and/or the editor(s). MDPI and/or the editor(s) disclaim responsibility for any injury to people or property resulting from any ideas, methods, instructions or products referred to in the content.
